# The Adaptability of Life on Earth and the Diversity of Planetary Habitats

**DOI:** 10.3389/fmicb.2017.02011

**Published:** 2017-10-16

**Authors:** Dirk Schulze-Makuch, Alessandro Airo, Janosch Schirmack

**Affiliations:** ^1^Astrobiology Group, Center for Astronomy and Astrophysics, Technical University Berlin, Berlin, Germany; ^2^Beyond Center, Arizona State University, Tempe, AZ, United States; ^3^School of the Environment, Washington State University, Pullman, WA, United States

**Keywords:** extremophiles, habitability, solar system, exoplanets, extraterrestrial environment, ocean planets, desert planets

## Abstract

The evolutionary adaptability of life to extreme environments is astounding given that all life on Earth is based on the same fundamental biochemistry. The range of some physicochemical parameters on Earth exceeds the ability of life to adapt, but stays within the limits of life for other parameters. Certain environmental conditions such as low water availability in hyperarid deserts on Earth seem to be close to the limit of biological activity. A much wider range of environmental parameters is observed on planetary bodies within our Solar System such as Mars or Titan, and presumably even larger outside of our Solar System. Here we review the adaptability of life as we know it, especially regarding temperature, pressure, and water activity. We use then this knowledge to outline the range of possible habitable environments for alien planets and moons and distinguish between a variety of planetary environment types. Some of these types are present in our Solar System, others are hypothetical. Our schematic categorization of alien habitats is limited to life as we know it, particularly regarding to the use of solvent (water) and energy source (light and chemical compounds).

## Introduction

Life on Earth inhabits a broad range of environments and is so ubiquitous that there is hardly any environment near the surface of our planet where no life can exist. Nevertheless, the range of some physicochemical parameters on Earth exceeds the ability of life to adapt, for example with respect to high temperatures. Other parameters such as radiation or pressure remain well within the limits of life on Earth. An example is pressure in the deep oceans which does not seem to affect life’s abundance and diversity. Low water activity, on the other hand, appears to be one parameter which is close to the limit of biological activity, as evidenced by research done in the hyperarid Atacama Desert and Antarctica’s Dry Valleys (Davila and Schulze-Makuch, 2016; Goordial et al., 2016). This does not mean, however, that all localities are habitats, places where life is actively metabolizing and reproducing. In many localities life simply survives in a dormant state, waiting for environmental conditions to change and become more suitable. Thus, when addressing the physicochemical limits of life on Earth, there is a need to distinguish between physicochemical limits that, if breached, destroy life (e.g., high temperature) and those under which life’s activity is terminated, but the organism is capable of surviving (e.g., low temperatures, desiccation).

Life also exists in an amazing diversity on Earth and this despite that all life on Earth has fundamentally the same biochemistry. While archaea and bacteria are mostly the record holder for adapting to a particular kind of extreme environmental stress or a combination of stresses, there are also eukaryotic organisms, even relatively complex animals and plants that can withstand or even metabolize and reproduce in harsh environments. Examples are tardigrades, which are known for their radiation and temperature tolerance, the Crucian Carp, which can slow its metabolism as an adaptation to low oxygen levels, and resurrection plants, which are specially adapted to desiccation (Islam and Schulze-Makuch, 2007). In assessing life’s ability to cope with extreme environmental conditions, we also need to differentiate between the limits of single organisms/cells versus single- and multiple-species communities, since their ability to adapt can change substantially. A particularly intriguing case are lichens, which are symbiotic organisms composed of phototrophs (algae or cyanobacteria) and fungi. This symbiosis makes lichens more resistant to combinations of environmental stresses compared to the individual phototrophs or fungi.

While the diversity of environmental niches and life forms on Earth is impressive, there is likely a much greater diversity of habitats existing on other planets within and especially outside our Solar System, particularly considering their projected immense quantity, with currently more than 3000 exoplanets confirmed and another 5000 awaiting confirmation. Many of the newly discovered planets have no analog in our Solar System such as “Super Earths”, presumed terrestrial rocky planets with a mass several times that of Earth.

It can be assumed that a subset of these alien environments will in principle be habitable for some organisms we know on Earth; however, the range of habitable niches could be even much greater for alternative alien biochemistries, which allows one to conjecture that life in the Universe would exhibit a much larger variety of forms and functions than life on Earth (Schulze-Makuch, 2015). Furthermore, if one considers other molecules than water as a solvent for an alien biochemistry, the types of possible habitats becomes highly speculative and increases enormously in quantity and therefore goes beyond the scope of this paper.

Here we review the adaptability of life, especially regarding to temperature, pressure, and water activity. We use this knowledge to outline the range of possible habitable environments on alien planets and moons based on life as we know it on Earth.

## Physicochemical Limits of Life

### Adaptation to Temperature Extremes

Life on Earth is based on carbon containing molecules as the major building blocks for biomass, water as a solvent and exergonic chemical reactions or light. These are energy sources that are life-sustaining and suitable for Earth (Schulze-Makuch, 2015), a terrestrial planet with a mean surface temperature of 15°C at an atmospheric pressure of 1 bar. Yet, the conditions under which life can persist on Earth are incredibly broad, also in regard to temperature range. Relatively, well-explored is the upper temperature limit of life. The current hyperthermophilic record holder, *Methanopyrus kandleri*, can grow at 122°C (Takai et al., 2008). The survival range is even higher, at least 130°C, as shown for *Geogemma barossii* (Kashefi and Lovley, 2003). Even if yet undiscovered species can thrive under higher temperatures, it can be assumed that no organism on Earth can cope with substantially higher temperatures, since the molecular building blocks of life as we know it, such as DNA, thermally disintegrate above about 150°C under wet conditions (White, 1984; Madigan and Orent, 1999). Conceivably, hyperthermophilic microorganisms could rapidly re-synthesize amino acids such as cysteine and glutamic acid that decompose quickly at higher temperatures, as well as low molecular weight compounds (e.g., NAD and ATP) that hydrolyze rather rapidly (Stetter, 1999). While the DNA within a cell is generally negatively supercoiled, all hyperthermophilic microorganisms studied so far have a reverse gyrase that positively supercoils the DNA (Baross et al., 2007). Daniel et al. (2004) pointed out that the thermal stability of DNA is further increased by the supercoiling when cationic proteins are present. Advantageous are also ether-linked, heat resistant lipids, which are commonly found in hyperthermophilic archaea and some hyperthermophilic bacteria. Beeby et al. (2005) suggested that the increased use of disulfide bonds and amino acid substitutions at high temperatures stabilizes protein structures. The formation of higher-order oligomers, the increase in ion-pair content, and the use of salts of various valence states demonstrates that the proteins of thermophiles have evolved to deal with the higher temperatures, which also enhances the stability of RNA and DNA (Rothschild and Mancinelli, 2001). Holden and Baross (1995) proposed that thermal stability of organic macromolecules is also increased by high pressure conditions such as usually existing at submarine hydrothermal vents, particularly when temperatures are so high that they approach the limit of life. The previous discussion does not only pertain to microorganisms. There are also thermophilic multicellular organisms. The Pompeii worm is one of the outstanding examples of a thermophilic metazoan, because it can withstand temperatures approaching 105°C (Chevaldonné et al., 1992). It does so by using both biochemical and physical means. For example, the worm uses the most thermostable fibrillar collagen known and it circulates cold water over its exterior body.

The lower temperature limit for active life is much less well-explored, since the metabolic activity in principle only ceases entirely, when the aqueous cell plasma freezes. Due to intracellular anti-freeze proteins, metabolic activity can occur well below 0°C and microbial growth has been determined for *Planococcus halocryophilus* down to -15°C (Mykytczuk et al., 2013). Recently, multicellular eukaryotes such as the lichen *Umbilicaria* and the yeast *Rhodotorula glutinis* have been shown to still grow at -17 and -18°C, respectively (De Maayer et al., 2014). However, since metabolic reaction rates are temperature dependent (described by the Arrhenius equation) metabolism and growth become so slow that experimental detection is increasingly difficult. Although, there have been reports of metabolic activity in permafrost soils at -39°C (Panikov et al., 2006), thermodynamic considerations suggest that at -40°C the metabolic turnover of the entire cellular carbon takes 100 million years (Price and Sowers, 2004). Hence, the determination of the lower temperature limit of life on Earth becomes experimentally impossible due to ever decreasing metabolic rates. Clarke et al. (2013) predicted that the limit for growth of psychrophilic organisms is approximately -26°C for microbes and -50°C for multicellular organisms with advanced organismal adaptations for thermoregulation based on the energetics of metabolic processes. As long as an organism is able to endure the process of freezing and thawing, there is no principle lower temperature limit for survival, since being in a frozen state is equivalent to being dormant. As shown for tardigrades the limit of survival can extend for some species to nearly absolute zero (Jönsson et al., 2008).

While the temperature range from about -40 to +150°C seems to represent the physicochemical limits of active life (with its ability to metabolize and grow) it can be speculated that organisms could be adapted to more extreme values, particularly higher temperatures, if a different biochemistry of putative alien organisms is assumed. However, even any alien organism will be bound to its limits of metabolism and reproduction, because of biochemical and energetic constraints. In principle, life cannot flourish above the critical temperature at which the solvent for life cannot remain anymore in the liquid phase. For water, this value is 374°C at 22 MPa pressure. If some other solvent is used by life such as ammonia, an ammonia-water mixture or methanol (e.g., Schulze-Makuch and Irwin, 2008), these limits will be different. In essence, the absolute limit depends on the major building blocks and the type of solvent used by life. Even if carbon-based building blocks and water pair up (as being the case on Earth), the biochemical diversity may be very different from life as we know it, including life’s physicochemical limits. In principle, even in this case the temperature range between 150°C and the critical point of water could still be accessed. The intriguing question is how far the envelope of life can be extended with a different type of biochemistry, and also different types of adaptations.

### Adaptations to Pressure Extremes

The physiological limit to pressure is not well-defined for life on Earth. The deepest regions of Earth probed to date seem not to be an obstacle for life. Microbes have been obtained from the Mariana Trench, which is 10,660 m deep with pressures reaching 110 MPa (Yayanos, 1995; Kato et al., 1998; Abe et al., 1999) and temperatures hovering around 2°C. Kato et al. (1998) showed that two species of bacteria apparently related to *Shewanella* and *Moritella* seem to be obligately barophilic, because they grow optimally at 70 MPa, but do not grow at pressures less than 50 MPa. Pledger et al. (1994) reported on archaea that were retrieved from localities near deep-sea hydrothermal vents and that survived at pressures of up to 89 MPa. It appears that the higher pressure at hydrothermal vents has a net effect of stabilizing organic molecules (Marion and Schulze-Makuch, 2006). Sharma et al. (2002) demonstrated with the help of a diamond anvil cell that *Shewanella oneidensis* and also some *Escherichia coli* strains can remain metabolically and physiologically active at pressures in the range of 68 to 1680 MPa for close to 30 h. In the pressure window of 1200 to 1600 MPa, viable bacteria were found in fluid inclusions within Ice VI crystals. One percent of the population stayed viable when pressure was reduced back to 1 bar (Sharma et al., 2002). The critical parameter in these experiments is the rate of pressure change, because organisms are very sensitive to abrupt pressure changes.

The lower limit of pressure for life is not well-determined either, but the main obstacle seems to be rather associated with desiccation and low water activity rather than pressure itself (Diaz and Schulze-Makuch, 2006). Schuerger and Nicholson (2016) exposed many different organisms to Mars surface pressure conditions of about 0.6 kPa, and showed that many species were surviving at this low pressure, even species not known as hypobarophiles. Thus, as a first working assumption we postulate that pressure conditions, at least as experienced on a terrestrial planet, such as Earth, will not constitute a major problem for alien life. Life on Earth seems to adapt relatively well to either high or low pressure conditions, and is only rather sensitive to sudden pressure changes.

### Adaptations to Low Water Activity

On Earth, water is the solvent critical to life without which organisms cannot survive. Its presence is so critical, because water provides an environment allowing some chemical bonds to be stable and to maintain macromolecular structure, while at the same time assisting in the dissociation of other chemical bonds. This occurs rather easily and therefore enables frequent chemical interchange and also transformations from one molecular state to another. The solvent is instrumental for dissolving many solutes, but at the same time is enabling certain macromolecules to resist dissolution. This, in turn, provides boundaries, interfaces, surfaces, and stereochemical stability. Further, the solvent’s density maintains critical concentrations of reactants and thus constrains their dispersal. The solvent is a medium in which biochemical reactions can operate by providing both a lower and upper limit to prevailing pressures and temperatures. The result is that the solvent funnels the evolution of metabolic pathways into a narrower range that is optimized for multiple interactions. And finally, the solvent is a buffer softening environmental fluctuations (Schulze-Makuch and Irwin, 2008). This kind of properties makes water essential for life’s activity. The parameter of water activity (a_w_) is usually used in this context to quantify whether a specific medium can provide enough free water to facilitate a microorganism’s cellular functions. The water activity of a fresh water lake is about 0.99 and very close to that of pure water of 1.0, because nearly all the water is available for an organism, while in a saturated sodium chloride solution (a_w_ ∼ 0.75) much of the water is bound to the hydration sphere of the salt ions, and thus not available for organisms. The lowest water activity measured for active metabolism of a microbial strain is 0.605, which has been reported for xerophilic fungi in a saturated sugar solution (Williams and Hallsworth, 2009). The lowest water activity measured at which halophilic archaea and bacteria were able to metabolize is 0.611, which has been tested in their natural habitat as well as in laboratory experiments, and could be extrapolated from measured growth curves (Stevenson et al., 2015a,b; Yakimov et al., 2015). However, water activities are often measured in bulk, e.g., using a large volume and thus can be misleading. Although a bulk water activity of a_w_ = 0.49 was measured in a liquid asphalt lake, active microorganisms were found in micro-droplets of water within the hydrocarbons (Meckenstock et al., 2014). Further, water activities fluctuate daily and seasonally, and organisms may be active once a certain threshold of a_w_ is breached. Stevenson et al. (2015b) suggested that there might be a common water activity limit of about 0.6 for all three domains of life on Earth based on its physicochemical constraints. The key for microorganisms not only to survive, but to remain active under desiccating environmental conditions with low water activity, is facilitated by the stabilization of enough liquid water within the cell or its direct vicinity. Therefore, two major adaption strategies have evolved among microorganisms.

One of the adaptions to desiccating conditions is the formation of extracellular polymeric substances (EPSs). The EPS serve to restrain the evaporation of water molecules in the direct cell surrounding or within cell accumulations (Tamaru et al., 2005; Anderson et al., 2012). Roberson and Firestone (1992) and Roberson et al. (1993) showed that the microstructure of EPS of *Pseudomonas* spp. cells did not change in shape from the normal to the desiccated state, but the EPS matrix was significantly larger and contained less protein. Also, the EPS matrix contained several times of the cells’ weight in water compared to the EPS of cells grown at high water activities. Desiccation did not appear to affect the activity of the tested cells, which was explained by a higher water retention potential of the EPS matrix (Roberson et al., 1993). The archaeon *Haloquadratum walsbyi* secrets halomucin, a 9159 amino acids long highly glycosylated and sulfated protein, believed to function analogous to EPS, protecting the cell from desiccation by forming a water-rich capsule (Stan-Lotter and Fendrihan, 2015).

**FIGURE 1:**
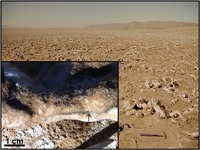
Yungay Salar, an analog environment for Mars. The insert on the lower left shows a salt crust populated by microorganisms using salt deliquescence for getting access to water. The dark band (arrow) within the salt crust is the pigment scytonemin.

Another adaption strategy is to attract as much water from the environment as possible rather than avoiding the loss of water under desiccating conditions. Davila et al. (2013) showed the use of salt deliquescence by microorganisms in halite nodules. The microbes were able to attract water molecules directly from the atmosphere in the Atacama Desert. This strategy is feasible when the relative humidity of the atmosphere was above 70%, equating to a bulk a_w_ > 0.7. This mechanism creates an ecological niche for microbial metabolism and growth in a hyperarid and otherwise hostile environment (**Figure 1**; Davila et al., 2008; Wierzchos et al., 2012). Jänchen et al. (2016) suggested that the deliquescence of a halite sample obtained from the Atacama Desert in combination with a hydrophilic biofilm formed by the cyanobacterium *Nostoc commune* might be a strategy for getting access to water for putative microorganisms living in environmental niches on Mars. The deliquescence effect of various salts was also implicated as a possible mechanism on Mars for staying longer habitable than previously thought, with the result that microbial communities may still be present in the southern highlands of Mars, where large salt deposits occur (Davila and Schulze-Makuch, 2016).

Areas of low water availability on Earth are often associated with high salt content; thus, organisms that live in desert environments, must be adapted to those as well. Examples are archaea and halophilic bacteria growing in 35% NaCl solution (Rodriguez-Valera et al., 1980; Antón et al., 2002; Asker and Ohta, 2002). Life at high salt concentrations is energetically expensive for microorganisms. The upper limit of physiological feasible salt concentrations is determined by bioenergetic constrains, as the amount of energy gained through the dissimilatory metabolism and how an organism adapts to osmotic stress (Oren, 2011). Any organism, which is capable to thrive in environments with high salt concentration must balance its cytoplasm osmotically to its surrounding medium. Two different basic strategies of adaptation exist: the first is based on the accumulation of ions in the cytoplasm. Mostly K^+^ instead of Na^+^ is used for this purpose with Na^+^ often being actively exported by Na^+^/H^+^ antiporters located in the cell membrane (Grant, 2004). This process is energetically not very cost intensive, but requires high salt concentrations for most of the cellular proteins to function properly and thus narrows down the adaptive range. This strategy is mainly used by a small group of halophiles (Oren, 2011). The second adaption strategy is the exclusion of salts from the cytoplasm and biosynthesis or accumulation of solutes at the same time, which are compatible with the cell machinery and therefore called compatible solutes (Borowitzka and Brown, 1974; Grant, 2004; Stan-Lotter and Fendrihan, 2013). The intercellular compatible solutes concentration is regulated to match the environmental salt concentration. Examples for compatible solutes are polyols like glycerol, sugars and sugar derivatives, betaines, ectoines and amino acids and their derivatives (Grant, 2004). This adaption process is energetically more cost intensive compared to the ion accumulation strategy mentioned above, but it offers a wider adaption range to different levels of salt concentrations (Oren, 2011).

Another often associated physical limit of life thriving in environments of low water availability on a planetary surface are increased levels of radiation, both ultraviolet (UV) and ionizing radiation. The most common form of harmful radiation on Earth’s surface is UV radiation acting frequently as a natural mutagen. Baross et al. (2007) pointed out that UV light can also prevent replication of cells, because of the dimerization of thymidine residues in the DNA. Ionizing radiation such as alpha and beta particles, gamma rays, and X-rays, and part of the ultraviolet spectrum can cause multiple breaks in the double-stranded DNA and is thus quickly detrimental to cells (Obe et al., 1992). Further, reactive oxygen species (ROS) are produced by ionizing radiation that can modify bases and/or cause single and double-strand DNA breaks (Rothschild and Mancinelli, 2001). Organisms evolved effective detoxification and inactivation mechanisms for ROS to counter their effect (Daly et al., 2004, 2007), and these mechanisms are positively correlated to higher internal Mn/Fe concentration ratios (>0.1) (Fredrickson et al., 2008). Most organisms on Earth have evolved protective mechanisms to counter harmful radiation. These include various DNA repair mechanisms and the accumulation of radiation-absorbing pigments. However, if an organism is resistant to one type of radiation is does not mean that it is resistant to some other type (Marion and Schulze-Makuch, 2006). *Deinococcus radiodurans* is best known for its resistance to ionizing radiation and can survive approximately 15,000 Gy, while *Thermococcus gammatolerans* can even survive 30,000 Gy (Hirsch et al., 2004). Mattimore and Battista (1996) suggested that this tolerance has evolved initially as an adaptation to desiccation. Leuko et al. (2015) claimed that experimental evidence indicates that halophilic adaptations may provide tolerance to radiation encountered in space. Fredrickson et al. (2008) argued that the UV and ionizing radiation resistance of soil bacteria is an incidental mechanism evolved to prevent oxidative protein damage induced during cycles of drying and rehydration, since most of the soil bacteria are shielded from such radiation by overlying soil. Surprisingly though, hyperthermophilic archaea recovered, e.g., from submarine hydrothermal vents have been found to withstand radiation levels of nearly 8,000 Gy (Jolivet et al., 2004; Beblo et al., 2011). Some multicellular organisms are also quite resistant to radiation such as the tardigrade *Ramazzottius varieornatus*. In its desiccated anhydrobiotic state it shows a high resistance to UVC (100–280 nm) (Horikawa et al., 2013), and the cockroach *Blattella germanica* can survive levels of ionizing radiation close to 1,000 Gy (Schulze-Makuch and Seckbach, 2013). Organisms limit radiation damage by a variety of microbial protection mechanisms. These include avoidance behavior, excision repair, photorepair, homologous recombinational repair, as well as the production of detoxifying enzymes and antioxidants (Yasui and McCready, 1998; Petit and Sancar, 1999; Rothschild, 1999; Smith, 2004). Other options for organisms to protect themselves from UV irradiation are habitation beneath protective layers of water or soil (Pierson et al., 1987; Wynn-Williams and Edwards, 2000), the development of iron-enriched silica crusts (Phoenix et al., 2001), self-shading (Smith, 1982), shielding by organic compounds that are derived from dead cells (Marchant et al., 1991), and the use of specialized organic pigments including scytonemin and carotenoids (Wynn-Williams et al., 2002). The major source of UV irradiation on Earth is the Sun, which is a yellow dwarf star (G2V). Due to Earth’s magnetic field the surface of our planet is relatively well-protected from ionic radiation. On an exoplanet however, the amount and spectrum of radiation it experiences at its surface will depend largely on the type and activity of the host star and whether the planet has a magnetic field.

## Planetary Environment Types (PETs) and their Habitability

In the following, we consider two major factors constraining the water-based habitability of planetary environment types (PETs): (1) high temperature, where the currently known limit is 122°C (Takai et al., 2008) and (2) low water activity, where the currently known limit is 0.6 (Williams and Hallsworth, 2009). Other factors influencing habitability, such as radiation, pressure, or water chemistry, can be mitigated, e.g., through shielding or adaptation and are not considered for PET classification.

**FIGURE 2:**
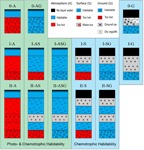
Planetary environment types (PETs) and their water-based habitability. Surface water is here defined as any substantial amount of liquid water residing on top of a solid surface (e.g., silicates, ice).

Here, we present a classification of PETs (**Figure 2**) centered on the habitability of three principle aqueous environments: (1) atmospheric water “A” (e.g., clouds/rain), (2) surface water “S” (e.g., morning dew or deep oceans), (3) and/or ground water “G”. The relevant factors constraining PET-habitability are temperature and water activity, which are largely dependent on planetary water content, size and age, as well as solar irradiation or the geothermal heat which mostly depends on the planetary composition and amount of radioactive elements. In the context of the here presented PET classification, we use the term ‘planet’ more broadly that includes moons and other planetary bodies.

Furthermore, we classify three compositional configurations based on the presence or absence of surface water layer(s): PET-0 lacks a surface water layer, while the other types possess different constellations of water layers. The absence of a surface water layer of PET-0 can have the following reasons: (1) The surface of the body lacks inherently water and is dry, which can be the result of a dry formation process or due to subsequent events or processes depleting an initial water content. (2) The atmospheric pressure is below the triple point value for water (<612 kPa), in which case liquid water is thermodynamically not stable. (3) The surface temperature is too low for liquid water to exist, which, if salts such as CaClO_4_ are present, can be as low as -70°C. (4) The surface temperature is too high at the respective pressure for water to condense.

PET-I and II have a surface water layer, ranging from a thin water film (e.g., morning dew) to massive Earth-like oceans or from water-ice caps to a global water-ice shell. PET-II is characterized by the presence of high-pressure water-ice, which is only possible if the planet is above a certain size and has a sufficiently massive water layer for generating the required pressures (e.g., 632 MPa at 273 K). While PET-I has only one liquid water layer, PET-II has two liquid water layers that are separated by an interstitial high-pressure water-ice layer. Finally, all PETs can host an atmosphere that permits the condensation of liquid water if the atmospheric density and temperature range are adequate.

Water-bearing PET sub-environments (atmosphere, surface, and ground) can be (1) uninhabitable due to the lack of liquid water (dry or frozen) or due to too high temperatures; (2) habitable, but not receiving any sunlight and therefore only permit chemotrophic life, or (3) habitable with access to sunlight allowing additionally phototrophic life to be possible.

Except for recurring slope lineae (RSL) and similar features on Mars, it is generally believed that Mars has no surficial liquid water, making it largely a PET-0 planet, where the polar water-ice caps would classify as PET-I. However, Mars is assumed to have a liquid subsurface water aquifer and consequently would be then classified a PET-0-G and PET-I-G, respectively.

If a PET-0 planet has a sufficient atmosphere for water to condense, microbial life is in principle possible within such air-born water droplets, as it has been suggested for the upper atmosphere of Venus (Schulze-Makuch et al., 2004, 2013), classifying it as PET-0-A. While on Venus the surface and subsurface is too hot for life to be possible, temperatures on other bodies can be low enough for stable liquid water at temperatures suitable for life in the subsurface. Such a PET would be classified as 0-AG and is exemplified by hyperarid deserts on Earth such as the Atacama Desert.

Earth has the greatest diversity of habitats in our Solar System occupying all habitat types (atmosphere, surface, ground) and hosts besides hyperarid deserts (0-AG), three types of PET-I: rivers, lakes, or oceans (I-ASG), continental ice such as the Greenland ice-cap (I-G) and ice-covered water bodies such as Lake Vostok, Antarctica (I-SG).

Under low surface temperatures or for smaller planets prohibiting a stable atmosphere, a surface water layer will be frozen as in the case for the global water-ice shell of Europa (I-SG). PET-II are characterized by a lower high-pressure water-ice layer underlain by a secondary ocean, which is only possible if the surface water layer (frozen and/or liquid) is sufficiently thick and the planet large enough for having high-pressure conditions at depth. For PET-II it is unclear whether the upper ocean layer is subject to nutrient limitation, since the interstitial high-pressure water-ice layer prevents a direct interaction with the silicate surface below. In the case of PET-II-AS/ASG, the upper ocean could receive direct nutrient input from cosmic dust. Such a nutrient input is not efficient for PET-II-S/SG, since the upper ocean is overlain by a water-ice crust.

## Discussion and Conclusion

The diversity of planets and moons in our Solar System is astonishing and considering the immense quantity of exoplanets the variability in the Galaxy is presumably even much larger. This is largely due to multiple factors affecting the nature of a planet, such as a planet’s size, composition, type of host star, formation scenario and orbital distance. Consequently, a geophysical-based comprehensive planet classification would need to be multi-dimensional and presumably would be highly complex. Also, planets are not static objects, but undergo an evolutionary path during which their habitability parameters can change dramatically. Over the past 4.5 billion years Earth has undergone fundamental changes such as the Moon-forming impact, the development of continental crust, Snowball Earth periods and the Great Oxidation Event. In the case of Mars and Venus, which both might have harbored Earth-like habitats early on, evolutionary changes have been even more dramatic (Schulze-Makuch et al., 2013) and resulted in a partial or possibly total loss of habitability.

While a few decades ago, there was much skepticism whether life could exist in the Solar System beyond Earth, multiple locations have today become sites for the search of life beyond Earth: Mars, Europa and Enceladus, as well as Ceres, Titan, and even Venus. This increase in conceivable potential habitats has led to multiple classification schemes being proposed.

Lammer et al. (2009) classified four planetary types, where their class-I planets are Earth-like planets that allow the evolution of complex multi-cellular life in contrast to class-II planets where life is possible but cannot develop complex forms, exemplified by Mars- and Venus-like bodies. The other two types are ocean-planets, where the ocean is in contact with the silicate surface (class-III) or not (class-IV). Noack et al. (2016) have focused on the classification of ocean-planets, where the H1 type hosts a single ocean layer that is in contact with a silicate surface, the H2 type has two ocean layers where the lower one is in contact with the silicate surface, and the H3 type with only one ocean that is not in direct contact with a silicate surface due to a high-pressure water-ice layer at the bottom.

We here present a classification of water-based PETs that are not necessarily occurring at a global scale and can occur in parallel on a single moon or planet. Although such a classification does not always allow the assignment of one PET to a single planetary body, it provides the possibility to describe the range of habitats on a single planet in more detail. While Lammer et al. (2009) classify Earth as a class-I planet we here require four PETs (0-AG, I-ASG, I-G, I-SG) to describe Earth’s habitat diversity. In fact, for an exoplanet to be truly Earth-like or being considered an Earth 2.0 planet, it would have to have multiple environmental habitats and the presence of a sizable biosphere and complex ecosystems, without which Earth, as we experience it, would not exist (Schulze-Makuch and Guinan, 2016). For planetary bodies with only one habitat type the classification is equivalent to other schemes; e.g., Europa is here classified as I-SG while Lammer et al. (2009) classifies such a body as class-III and Noack et al. (2016) as H1 with a surficial water-ice crust.

We have to emphasize that the PET scheme is based on life as we know it, which particularly applies to the use of solvent and energy source. If life’s biochemistry is or can be markedly different, for example, as a result of the utilization of a different type of solvent or energy source, this will also alter the results of the habitability assessment. A solvent with a lower liquidity range such as ammonia or an ammonia-water mixture would significantly decrease the temperature range at which life might be viable. If life can utilize a different source of energy such as magnetic fields, thermal or osmotic gradients (e.g., Schulze-Makuch and Irwin, 2008), then this may open up new habitats, that would otherwise not be viable. A test case of the biochemical diversity of life in our Solar System might be Titan, which is partly covered by liquid non-polar hydrocarbons (methane and ethane). If those hydrocarbons can function as a solvent of life, this would require a biochemistry different from the one we are familiar with. A non-polar solvent would mean that the membrane of cells, which on Earth have outer polar ends to interact optimally with a polar solvent (water), would not be suitable on Titan, but would likely have to be hydrophobic. Further, the extremely cold temperatures on the surface of Titan would likely require the utilization of chemical reactions energetic enough to overcome the otherwise low kinetics in this type environment. This might even mean the utilization of radical reactions (Schulze-Makuch and Grinspoon, 2005), which are in general much more energetic than the redox-reaction on which life on Earth is based on. Thus, the PET scheme as outlined above (**Figure 2**) has to be understood as a first attempt to generalize some of the habitats that are possible to support life, but it is unlikely to encompass all possibilities for life in the Universe.

## Author Contributions

DS-M: concept, acquisition, interpretation, drafting; AA: concept, acquisition, interpretation, drafting; JS: concept, acquisition, interpretation, drafting.

## Conflict of Interest Statement

The authors declare that the research was conducted in the absence of any commercial or financial relationships that could be construed as a potential conflict of interest.
